# Electrically tunable perfect light absorbers as color filters and modulators

**DOI:** 10.1038/s41598-018-20879-z

**Published:** 2018-02-08

**Authors:** Seyed Sadreddin Mirshafieyan, Don A. Gregory

**Affiliations:** 1 Department of Electrical and Computer Engineering, The University of Alabama in Huntsville, Huntsville, Alabama 35899 USA; 2 Department of Physics, The University of Alabama in Huntsville, Huntsville, Alabama 35899 USA

## Abstract

Methods for spectrally controlling light absorption in optoelectronic devices have attracted considerable attention in recent years. It is now well known that a Fabry-Perot nanocavity comprising thin semiconductor and metal films can be used to absorb light at selected wavelengths. The absorption wavelength is controlled by tailoring the thickness of the nanocavity and also by nanostructure patterning. However, the realization of dynamically tuning the absorption wavelength without changing the structural geometry remains a great challenge in optoelectronic device development. Here it is shown how an ultrathin n-type doped indium antimonide integrated into a subwavelength-thick optical nanocavity can result in an electrically tunable perfect light absorber in the visible and near infrared range. These absorbers require simple thin-film fabrication processes and are cost effective for large-area devices without resorting to sophisticated nanopatterning techniques. In the visible range, a 40 nm spectral shift can be attained by applying a reasonable bias voltage to effect the color change. It is also shown that these electrically tunable absorbers may be used as optical modulators in the infrared. The predicted (up to) 95.3% change in reflectance, transforming the device from perfectly absorbing to highly reflective, should make this technology attractive to the telecommunication (switching) industry.

## Introduction

Thin-film light absorbers have recently received considerable attention due to their straightforward fabrication, low cost, and wide range of potential applications, however they have been restricted to the near infrared range or they are not tunable, or they are not perfect absorbers. Tunable perfect light absorbers functioning in the visible range as discussed in this paper comprise a Fabry-Perot nanocavity made of thin metal and semiconductor films that absorb light completely over selected wavelength ranges in the visible and infrared. The fundamental absorption wavelength is determined by the thickness of the nanocavity and tunability is bias voltage controlled. This newest generation of perfect light absorbers would have many interdisciplinary applications in chemical and biological sensing^1–7^, solar energy harvesting^8–11^, photodetectors^12,13^, gas sensors^14^, structural color printing^15–18^, and color filters^19–26^. However, if the absorption could be controlled in real time, then multiple new applications can be envisioned such as high speed, high resolution, high grey scale displays, smart windows, and a variety of telecommunication devices to compete with those currently available. To ascertain the possibilities, a theoretical investigation into ultrathin spectrally selective perfect light absorption in a nanocavity structure made of an epsilon-near-zero (ENZ) material (as the active layer) is summarized here. The metal/ENZ/dielectric/metal structure is modeled as a nanocavity, allowing the enhancement of light absorption at the resonant wavelength to be explored. The ENZ material investigated here is a doped semiconductor whose carrier density can be controlled by an applied electric field bias, leading to the optical absorption becoming electrically tunable.

Unpatterned thin-film layer structures are typically made of multilayer thin dielectric and metal films. N-type doped indium antimonide (n-InSb) as the active ENZ layer in a metal/ENZ/dielectric/metal cavity structure is investigated because of its potential applications in a variety of technical fields. The metal-semiconductor-metal structure forms a Fabry-Perot cavity which traps incident light at the resonant wavelength. Enhanced light absorption occurs because of strong optical interference in the semiconductor film. In unpatterned thin-film layer structures the peak absorption wavelength can be tuned from the UV to the visible^6,19^ and into the mid-infrared^27^ regions by changing the thickness of the semiconductor layer. However, a technique for spectrally selecting the absorption by applying an electric potential across the structure would be a major advancement in the field.

Epsilon-near-zero materials are doped semiconductors whose real permittivity value slowly changes sign from positive to negative with increasing wavelength^28,29^. These materials, such as indium tin oxide (ITO) and n-InSb, are already widely used in the semiconductor industry^30–32^. ITO, for example, is often used as a transparent conductive layer in display applications. ENZ materials have also received considerable attention as potential electro-optical devices. Researchers have demonstrated narrow band^33,34^ and broad band^29^ directional perfect light absorption in nanocavity devices made of ENZ materials for transverse magnetic (TM) incident light polarization. An ENZ material (specifically ITO) has also been used in creating electrically tunable absorbers in the infrared regime^35–37^ and nonlinear optical devices^38^. In the visible range however, achieving electrically tunable perfect light absorption still remains a significant challenge. These electrically tunable absorbers are of great interest in many applications from color filters and high resolution displays to photodetectors and biosensors, and even in decorative arts and real-time tunable color coatings.

In recent publications, spectrally selective perfect light absorption in Fabry-Perot nanocavities has been experimentally demonstrated by varying the thickness of the cavity layer^6,19^, thermal annealing^19^, and altering the metallic substrate^22^. To dynamically tune the optical absorption without changing the structure geometry, J. Park *et al*.^35^ and F. Yi *et al*.^36^ have recently demonstrated electrically tunable infrared optical absorbers using ENZ material (ITO) in grating plasmonic structures. Y. Yao *et al*.^39^ also showed that electrically tunable absorption can be realized using graphene in a plasmonic structure. However, in such structures the absorption was not complete and the fabrication process was complex due to the required nanostructure patterning. Indeed, the absorption occurred in the infrared region, not in the visible. Therefore, the structures cannot be used for color filters applications. In this work, simple thin-film structures (without nanopatterning) in conjunction with an ultrathin n-InSb film as an active ENZ layer in an optical nanocavity are proposed and modeled. Results indicate that voltage tunable perfect light absorption in the visible regime (without changing the structural geometry) should be possible.

## Drude Model

For the present study, the optimum ENZ material should have a real permittivity near zero in the visible or infrared region. The wavelength can be adjusted by changing the carrier density of the material via controlling the deposition conditions^19^ and doping level^40^. ENZ materials afford significant variation in their optical properties (i.e., permittivity and refractive index) due to their dependence on carrier depletion or accumulation^37^. By applying a negative voltage to the ENZ layer, an accumulation region can be created inside the ENZ layer (at the boundary with an underlying dielectric layer), causing the carrier density in the ENZ material to increase, as will be discussed later. Changing the carrier density in the ENZ material results in a change in its permittivity and consequently its refractive index, according to the classic Drude model^41–44^:

1 $$\varepsilon =\varepsilon ^{\prime} -j\varepsilon ^{\prime\prime} ,\quad \varepsilon ^{\prime} ={\varepsilon }_{\infty }-\frac{{\omega }_{p}^{2}}{{\omega }^{2}+{\gamma }^{2}},\quad \varepsilon ^{\prime\prime} =\frac{\gamma {\omega }_{p}^{2}}{\omega ({\omega }^{2}+{\gamma }^{2})},$$

2 $${\omega }_{p}=\sqrt{\frac{N{e}^{2}}{{m}^{\ast }{\varepsilon }_{0}}},\quad {\omega }_{ENZ}=\sqrt{\frac{{\omega }_{p}^{2}-{\varepsilon }_{\infty }{\gamma }^{2}}{{\varepsilon }_{\infty }}},$$

3 $$n=n^{\prime} -jn^{\prime\prime} ,\,\,\,\,\,n=\sqrt{\varepsilon },$$

where *ε′* is the real part and *ε″* is the imaginary part of the complex permittivity *ε*. *ε*_∞_ is the permittivity at high frequencies, *ω* = *2πc/λ*, *γ* is the scattering constant, *ω*_*p*_ is the plasma frequency, and *ω*_*ENZ*_ is the ENZ frequency when *ε′* = 0. In equation (2), *N* is the carrier density, *e* is the electron charge, m* is electron effective mass, and *ε*_0_ is the permittivity of vacuum. In equation (3), *n* is the complex refractive index of the ENZ material with a real part of *n′* and an imaginary part of *n″*.

Some metals, doped semiconductors, and metamaterials^45,46^ can be ENZ materials as well, with dielectric constants adequately described by the Drude model^47^. Doped semiconductors have metal-like behavior in the infrared region similar to those of metals in the UV-visible range. For metals, *ε*_∞_ = 1 and *γ* ≪ *ω*_*p*_, and therefore the ENZ wavelength *ω*_*ENZ*_, where the real part of the permittivity vanishes, coincides with the plasma frequency *ω*_*p*_ according to the Drude model. For doped semiconductors however, the plasma frequency *ω*_*p*_ and the ENZ frequency *ω*_*ENZ*_ are different. In this study, n-InSb (tellurium doped indium antimonide) is chosen as the ENZ material, with an achievable electron carrier density of 3.5 × 10^17^ cm^−3^ and a resulting ENZ wavelength of 35.17 µm^40^. Optical properties of n-InSb are listed in Table 1. This material is a good candidate for electron carrier density modulation induced by an electric voltage due to its low electron effective mass of 0.023*m*_*e*_ compared to other common ENZ materials such as ITO with an effective mass of 0.4 *m*_*e*_^37^. At a fixed plasma frequency, an ENZ material with a smaller effective mass has a smaller carrier density according to the Drude model. This is favorable for carrier density modulation because the carrier density of ENZ materials can be easily increased with an applied negative voltage. Many ENZ materials such as ITO and TiN have ENZ wavelengths in the near infrared and visible range due to their high electron carrier densities (*N* ~ 10^19^–10^21^ cm^−3^), which is close to that of metals, however it is difficult to further increase (or modulate) their carrier densities. On the other hand, n-InSb has a substantially lower electron carrier density of *N* ~ 10^17^ cm^−3^, making this material a suitable candidate for electrically induced carrier modulation, perhaps increasing the carrier density to that of some metals. Therefore, a large accumulation region can be created in the n-InSb layer, changing its optical properties dramatically.

**Table 1:** Measured optical properties of n-InSb^40^.

Material	*ε*_∞_	m*	*γ* (rad/s)	*N* (cm^−3^)	*ω*_*p*_ (rad/s)	*ω*_*ENZ*_ (rad/s)	*λ*_*p*_ (µm)	*λ*_*ENZ*_ (µm)
n-InSb	16.8	0.023*m*_*e*_^(a)^	2.1 × 10^12^	3.5 × 10^17^	2.20 × 10^14^	5.36 × 10^13^	8.57	35.17

^(a)^*m*_*e*_ = 9.11 × 10^−31^ kg (electron rest mass).

A material such as n-InSb, with a plasma frequency *ω*_*p*_ in the infrared, behaves like a dielectric in the visible range. With the application of a negative voltage, the electron carrier density of n-InSb increases and an accumulation region is created. As a result, the plasma frequency *ω*_*p*_ shifts toward the visible range according to the Drude model of equations (1) and (2). Hence the n-InSb material behaves as a metal in the thin accumulation region. Metals have a plasma frequency in the UV-visible range. This significant change in the optical properties of n-InSb paves the way for many potential applications in optoelectronic devices such as electrically tunable color filters and high resolution real-time displays.

## Absorber Structure

**Figure 1:**
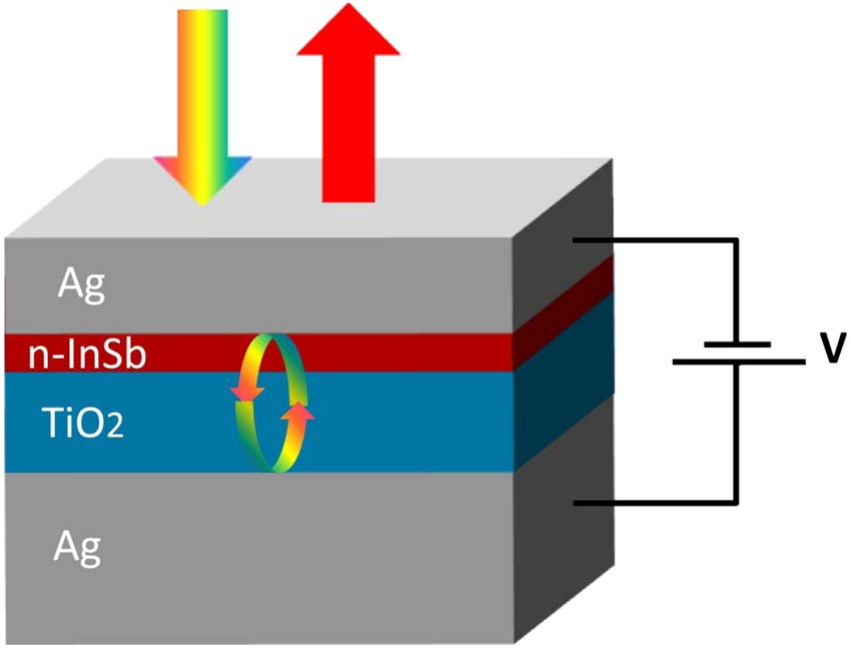
Schematic structure of an electrically tunable perfect light absorber.

The schematic of the proposed electrically tunable perfect absorber is shown in Fig. 1. The device consists of Ag/n-InSb/TiO_2_/Ag thin-film layered structure. The complete structure forms a Fabry-Perot cavity which absorbs light completely at its resonance wavelength. N-type indium antimonide (n-InSb) and titanium dioxide (TiO_2_) together form a cavity medium sandwiched by two silver (Ag) mirrors. The bottom Ag layer is 100 nm (optically thick) with no transmission and the top Ag layer is 35 nm thick. The top and bottom Ag mirrors have approximately 97% and 90% reflectance in the visible range, respectively. The n-InSb layer is 10 nm thick and the thickness of the TiO_2_ layer is 40 nm. The absorption wavelength of the structure depends on the thickness of the cavity medium (n-InSb + TiO_2_). In this configuration, the device has an absorption wavelength in the visible range, perfect for color filter applications. If an electric voltage is applied to the structure, then the observed color of the device changes accordingly. In Fig. 1, the large dc dielectric constant of the TiO_2_ layer underneath the n-InSb layer increases the structure’s breakdown voltage^48^.

**Figure 2:**
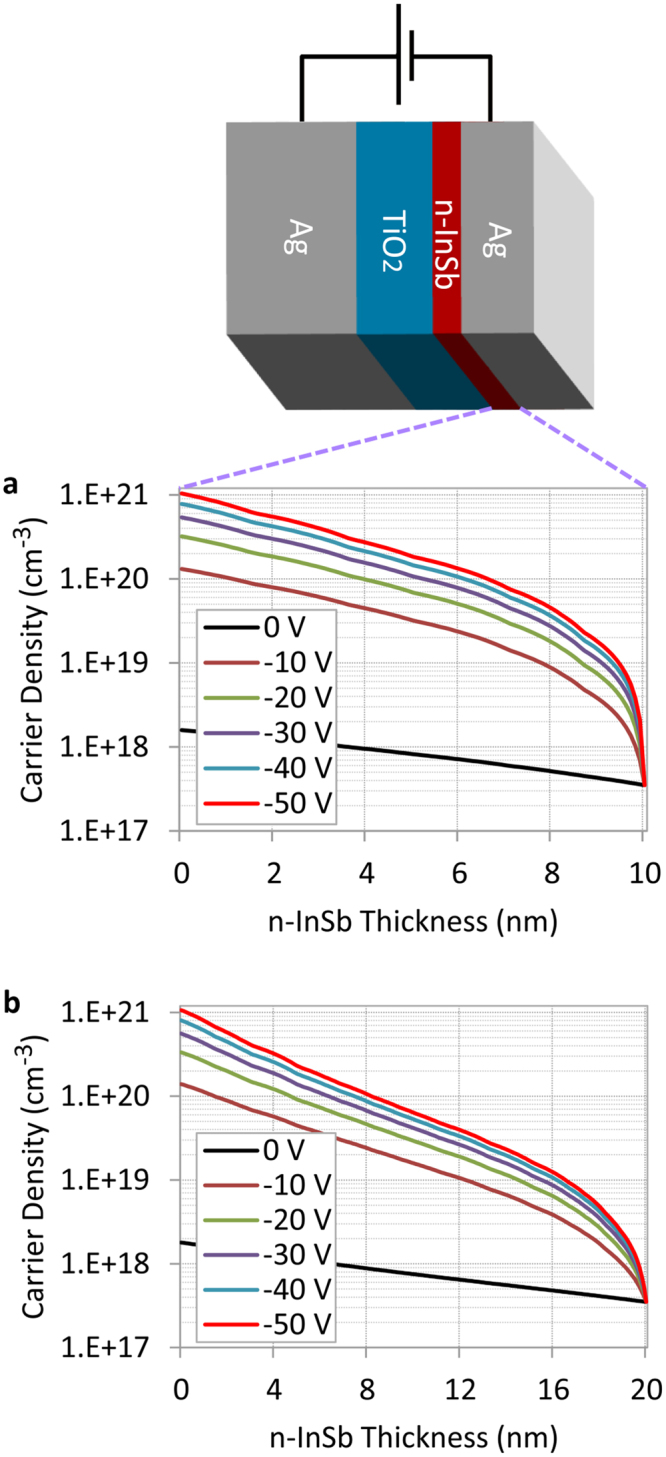
Modulation of electron carrier density inside the n-InSb films with thicknesses of (**a**) 10 nm, and (**b**) 20 nm with the application of a bias voltage. By applying a negative voltage, an accumulation region is created inside the InSb film.

The charge distribution across the n-InSb layer is calculated using Lumerical DEVICE software® with the inclusion of a bias voltage. Required parameters include the dc dielectric constant of TiO_2_ (approximately 80^48^) and the work function of silver (approximately 4.26 eV^49^). For InSb, the bandgap was taken to be: 0.17 eV^50^, the dc dielectric constant: 16.8^51^, and the work function: 4.77 eV^52^. Figure 2 shows the simulation results for 0 V to −50 V. n-InSb has a carrier density of *N* = 3.5 × 10^17^cm^−3^ when there is no voltage. By applying a negative voltage to the top Ag surface, an accumulation layer is created inside the n-InSb layer and the electron carrier density is increased to *N* = 10^21^ cm^−3^ (for −50 V at the boundary with the TiO_2_ layer), as shown in Fig. 2. Simulations for two devices with different n-InSb thicknesses (10 nm and 20 nm) were done. These two devices are predicted to have different absorption wavelengths in the visible range.

**Figure 3:**
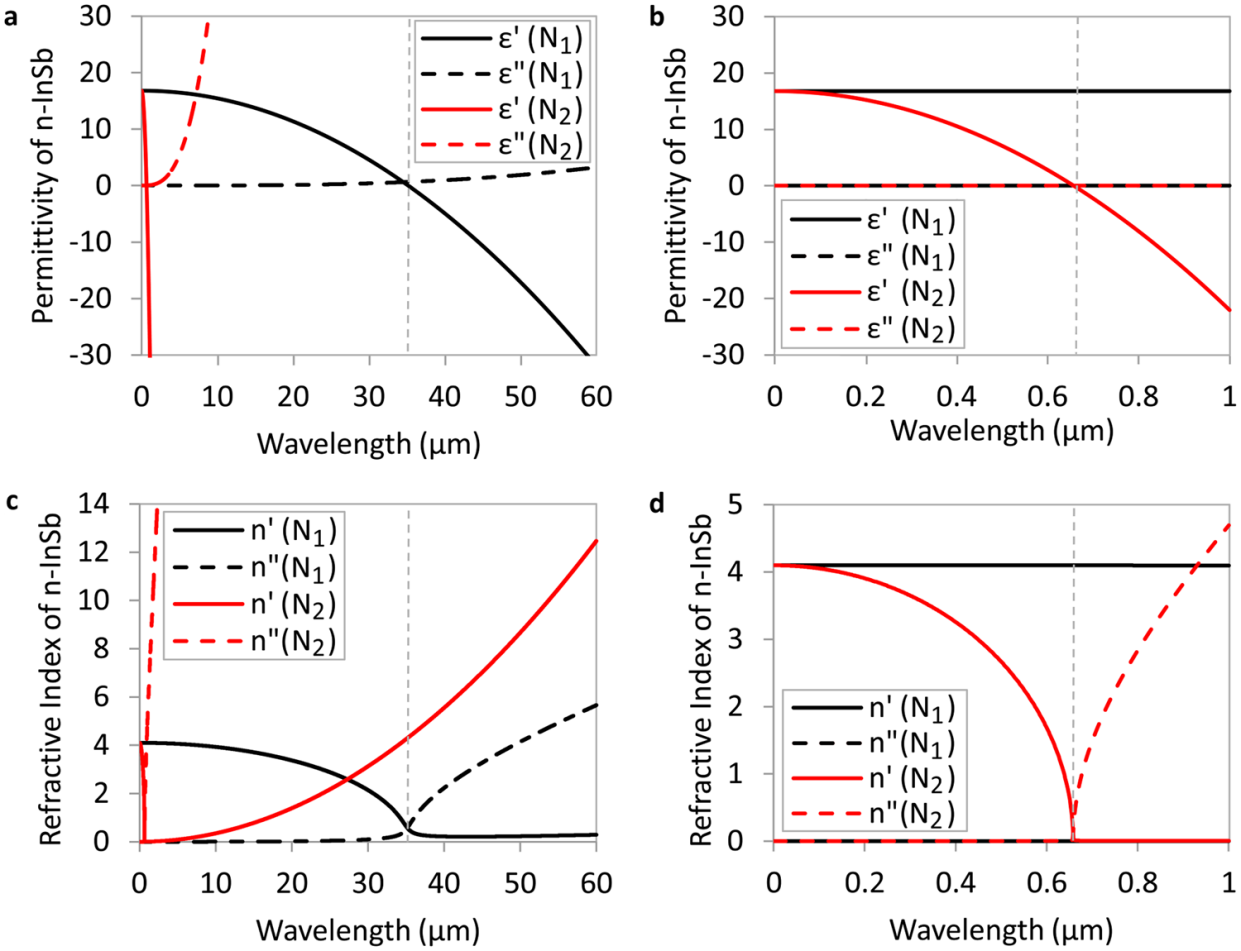
(**a**,**b**) Permittivity, and (**c**,**d**) refractive index of n-InSb for *N*_1_ = 3.5 × 10^17^ cm^−3^ and *N*_2_ = 10^21^ cm^−3^ in the infrared (**a**,**c**) and visible (**b**,**d**) regime according to the Drude model.

The dielectric constant and refractive index of n-InSb films with carrier densities of *N*_1_ = 3.5 × 10^17^ cm^−3^ and *N*_2_ = 10^21^ cm^−3^ are calculated using the Drude model of equations (1)–(3). The results are shown in Fig. 3. For 0 V (*N*_1_ = 3.5 × 10^17^ cm^−3^), *λ*_*ENZ*_ occurs in the infrared at a wavelength of 35.17 µm. By applying a voltage of −50 V, the electron carrier density of n-InSb increases to *N*_2_ = 10^21^ cm^−3^ at the interface with TiO_2_, and *λ*_*ENZ*_ shifts to a shorter wavelength in the visible, to 657 nm. As shown in Fig. 3b, in the visible range (which is the region of interest for color filter applications), the real part of the permittivity of n-InSb, *ε′*, is constant for carrier density *N*_1_ which is the behavior of a dielectric. However, for carrier density *N*_2_, the real part of the permittivity of n-InSb changes from positive to negative with wavelength, which is a metallic behavior. This significant change in the permittivity of n-InSb with corresponding dielectric to metal-like behavior change yields a sizeable variation in the material’s refractive index, as shown in Fig. 3d. The absorption in the structure (Fig. 1) directly depends on the refractive index of the n-InSb layer, as will be discussed later. Thus, an electrically tunable absorber may be possible.

**Figure 4:**
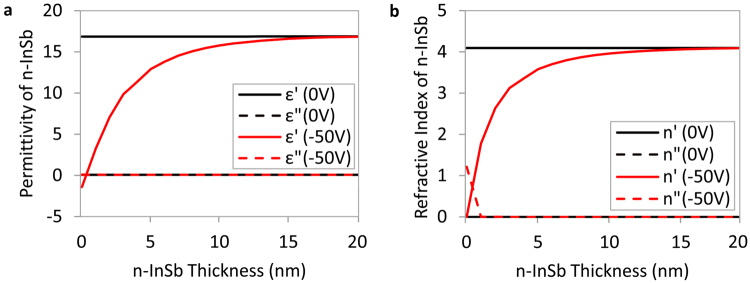
Variation of (**a**) permittivity and (**b**) refractive index inside the n-InSb layer at the absorber resonance wavelength of 665 nm due to the application of a bias voltage.

When a negative voltage is applied to the structure, the electron carrier density inside the n-InSb layer is increased exponentially and the maximum density occurs at the boundary with the TiO_2_ (see Fig. 2). At 0 V, the device with a 20 nm thick n-InSb has a resonance wavelength of 665 nm in the visible range. The variation of permittivity and refractive index inside the n-InSb layer at this resonance wavelength is calculated using the Drude model and shown in Fig. 4. As can be seen, when no voltage is applied, the permittivity and refractive index of n-InSb remain constant across this layer at the resonance wavelength. This is due to the ENZ wavelength of the n-InSb being in the infrared (35.17 µm) and thus constant in the visible range (see Fig. 3b). The visible range is of particular interest because in this range spectrally selective absorption results in optical colors. By applying an electric voltage of −50V, a dramatic change in permittivity and refractive index of n-InSb is observed in the visible range as shown in Fig. 4. This change is maximum at the boundary between n-InSb and TiO_2_, as expected, due to the maximum induced carrier density in this region (see Fig. 2).

## Simulation Results

In the absorber structure shown in Fig. 1, the bottom silver film is optically thick with no transmission, so that the optical absorption in the structure is *A* = 1 − *R*, where *R* is the optical reflectivity of the structure. The optical reflectivity *R* can be calculated using the transfer matrix method^53^

4 $$R={|r|}^{2}={|\frac{{M}_{21}}{{M}_{11}}|}^{2},$$

where *M*_11_ and *M*_21_ are the elements of matrix *M*,

5 $$M=[\begin{array}{cc}{M}_{11} & {M}_{12}\\ {M}_{21} & {M}_{22}\end{array}]={D}_{1}^{-1}({D}_{2}{P}_{2}{{D}_{2}}^{-1})({D}_{3}{P}_{3}{{D}_{3}}^{-1})({D}_{4}{P}_{4}{{D}_{4}}^{-1}){D}_{5},$$

6 $${P}_{i}=[\begin{array}{cc}exp(j{\phi }_{i}) & 0\\ 0 & exp(-j{\phi }_{i})\end{array}],\quad {\phi }_{i}=\frac{2\pi (n{^{\prime} }_{i}-j{n^{\prime\prime} }_{i}){d}_{i}}{\lambda }.$$

For TE polarization,

7 $$\,{D}_{i}=[\begin{array}{cc}1 & 1\\ (n{^{\prime} }_{i}-j{n^{\prime\prime} }_{i})cos{\theta }_{i} & -(n{^{\prime} }_{i}-j{n^{\prime\prime} }_{i})cos{\theta }_{i}\end{array}],$$

and for TM polarization,

8 $${D}_{i}=[\begin{array}{cc}cos{\theta }_{i} & cos{\theta }_{i}\\ (n{^{\prime} }_{i}-j{n^{\prime\prime} }_{i}) & -(n{^{\prime} }_{i}-j{n^{\prime\prime} }_{i})\end{array}].$$

**Figure 5:**
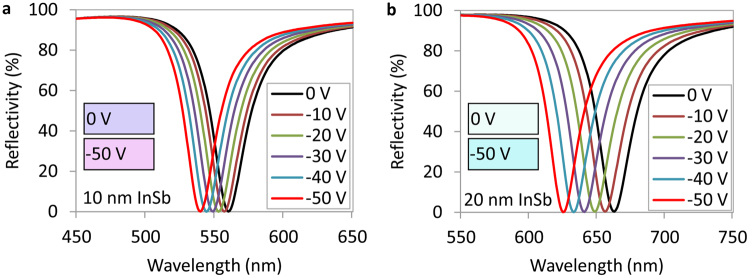
Calculated reflectance of two tunable perfect light absorbers with n-InSb thicknesses of (**a**) 10 nm, and (**b**) 20 nm. The thicknesses of top Ag, TiO_2_, and bottom Ag are 35 nm, 40 nm, and 100 nm, respectively. The reflectivity is calculated at normal incident angle. The subfigures illustrate the predicted colors of the devices under 0 V and −50 V.

In the equations above, *i* = 0, 1, 2, 3, 4 represents air, the top Ag layer, n-InSb, TiO_2_, and the bottom Ag layer, respectively. *θ*_*i*_ is the incident angle in the medium *i*, and *n*_*i*_′ and *n*_*i*_″ are the real and imaginary parts of the complex refractive index *n*_*i*_ of medium *i*. The refractive index of n-InSb is calculated from the Drude model and the refractive indices of Ag and TiO_2_ are taken from Palik^54^. Since the refractive index of n-InSb varies with the depth of the film (see Fig. 4b), the n-InSb layer (10 nm and 20 nm) is divided into 1 nm-thick sublayers with an approximately constant refractive index for each sublayer in the transfer matrix calculations. *φ*_*i*_ is the propagation phase delay of the optical wave in medium *i*. In equation (6), *d*_1_ is the thickness of the top Ag layer (35 nm), *d*_2_ is the thickness of the n-InSb layer (10 nm and 20 nm), and *d*_3_ is the thickness of the TiO_2_ layer (40 nm). Using the transfer matrix method, the reflectivity of the structure is calculated and shown in Fig. 5. The thickness of n-InSb in Fig. 5a is 10 nm and 20 nm in Fig. 5b. As a result, at 0 V, the resonance wavelengths of the absorbers with 10 nm and 20 nm thick n-InSb layers are 560 nm and 665 nm, respectively. These devices reflect different colors as illustrated in Fig. 5a and b. Complete light absorption is seen in these devices at their resonance wavelengths. The fundamental perfect light absorption wavelength can be easily adjusted in the visible and infrared ranges by changing the thickness of the InSb layer.

With the application of a negative voltage of −50 V to the structure, a spectral shift of 20 nm is observed for the device with a 10 nm n-InSb layer (see Fig. 5a), and a 40 nm spectral shift is seen for the device with a 20 nm thick n-InSb layer, as shown in Fig. 5b. Consequently, the reflected colors of both devices are expected to change with applied voltage. By increasing the thickness of the n-InSb layer, the absorption wavelength shifts toward longer wavelengths, but doing so also provides a larger modulation (40 nm shift for a 20 nm InSb device vs. 20 nm shift for a 10 nm InSb device; both with the application of the same −50 V bias) due to the creation of a larger accumulation region inside the InSb layer. The device with a 20 nm InSb layer has a shift twice that of the device with a 10 nm InSb because, for the same −50 V, the number of electron charges injected into the 20 nm InSb layer is twice that of the 10 nm InSb layer, simply due to its doubled thickness (twice as many electrons available). Therefore, the accumulation region in the 20 nm device is double that of the 10 nm InSb device (see Fig. 2). A larger accumulation region allows a greater modulation of the permittivity and refractive index of n-InSb, which results in a larger shift in the absorption spectrum as shown in Fig. 5b. The 20 nm InSb device is near the optimum design for modulation in the visible spectrum, which is our region of interest for color filter applications. Further increasing the thickness of InSb would shift the absorption wavelength toward the infrared. In Supplementary Fig. S1 it is also shown that for −50 V, if the InSb thickness is increased to more than 20 nm, the accumulation region does not increase significantly, indicating that 20 nm InSb is near the optimum design, and showing that we cannot continue to double the thickness and expect a further doubling of the modulation. The limit of our architecture is determined by the breakdown voltage, which is −60 V. Larger accumulation regions can be achieved if we increase the thickness of the bottom TiO_2_ layer to increase the breakdown voltage, however the absorption wavelength will also shift to the infrared, as will be shown later. These results are promising and may lead to fabricating devices operating in real-time that can manipulate light absorption with a corresponding reflected color change.

## Optical Modulators

**Figure 6:**
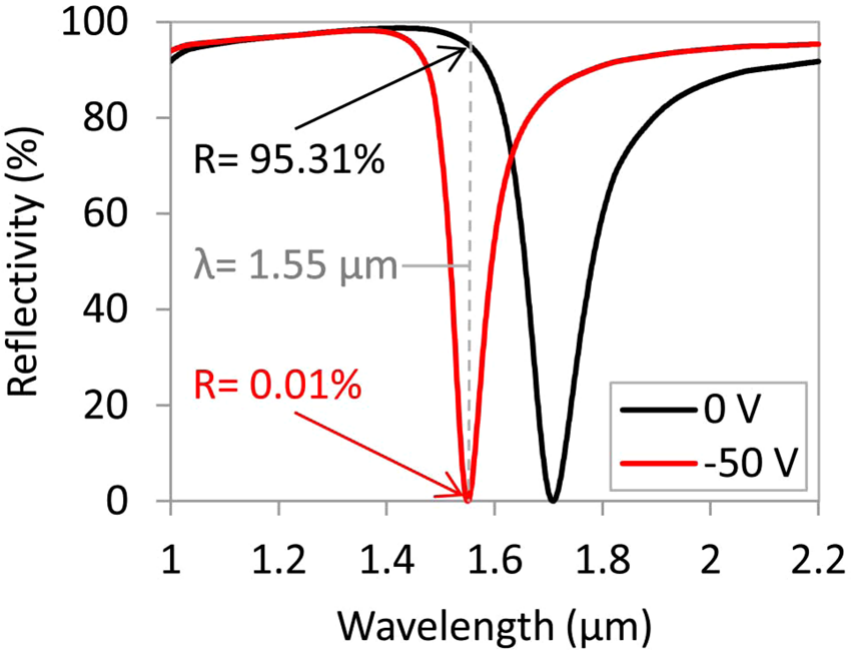
Calculated reflectivity of the tunable perfect light absorber composed of Ag/n-InSb/TiO_2_/Ag multilayer structure with corresponding thicknesses of 18 nm/40 nm/215 nm/100 nm, when it is unbiased (black) and biased (red) with −50 V.

An electrically tunable perfect light absorber could also be used for telecommunications switching applications. By adjusting the thickness of the absorber structure in Fig. 1 to 18 nm for the top Ag layer, 40 nm for the n-InSb layer, 215 nm for the TiO_2_ layer, and 100 nm for the bottom Ag layer, the peak absorption wavelength at −50 V will be near the telecommunication wavelength of 1550 nm in the infrared regime. At this wavelength, the reflectivity of the structure is *R* = 0.01% and the absorption is complete (*A* = 99.99%). By reducing the voltage to 0 V, the peak absorption wavelength shifts toward the red, and the reflectivity from the structure at λ=1550 nm increases to *R* = 95.3%, where the absorption is only *A* = 4.69% as shown in Fig. 6. By reducing the voltage from −50 V to 0 V, the perfect absorption wavelength moves from 1550 nm to 1710 nm, a 160 nm spectral shift. The absolute reflectance change of this structure is 95.3% which is much higher than other modulators recently reported with only 15%^35^ reflectance change. In addition, the normalized modulation at the wavelength of 1550 nm is *ΔR* = *R* (−50V)/*R* (0 V) = 9531% which is significantly higher than the 35%^35^ reported in the literature.

## Conclusions

In this work, an investigation into electro-optically tunable perfect light absorbers based on epsilon-near-zero materials is presented. The absorber structure comprises unpatterned thin-film metal and semiconductor materials, forming a Fabry-Perot cavity that functions as a wavelength-selective light absorber. By properly choosing the cavity thickness, the absorption wavelength can fall in the visible range, and thus the reflected color of the device can be changed with applied voltage. n-InSb was chosen as the ENZ material for this investigation because of its electro-optic characteristics. By applying a negative voltage, the carrier density of n-InSb is increased and an accumulation region is created inside the n-InSb layer. This yields a dramatic change in the optical properties of the material, from a semiconductor-like behavior to a metal-like behavior in the accumulation region. A 40 nm spectral shift in the visible range with the application of −50 V to the structure is predicted, which would result in a broad spectral change in the color reflected by the device. The absorption wavelength can also be engineered to the 1550 nm telecommunication wavelength by adjusting the cavity layer thickness. At this wavelength, with the application of −50 V, a high modulation ratio (with a 95.3% change in reflectance) for the structure is predicted.

## Electronic supplementary material

Supplementary Information
