# Supplementary material for: Electrically tunable perfect light absorbers as color filters and modulators
Source: Sci Rep. 2018 Feb 8;8:2635. doi: 10.1038/s41598-018-20879-z (PMC5805698; doi:10.1038/s41598-018-20879-z)
Supplement: Supplementary file 1 — Supplementary Information [file 41598_2018_20879_MOESM1_ESM.pdf]

Supplementary Information for

Electrically tunable perfect light absorbers as color filters and modulators

Seyed Sadreddin Mirshafieyan[1] and Don A. Gregory[2,*]

[1]Department of Electrical and Computer Engineering, The University of Alabama in Huntsville, Huntsville, Alabama 35899, USA

[2]Department of Physics, The University of Alabama in Huntsville, Huntsville, Alabama 35899, USA

*E-mail: gregoryd@uah.edu

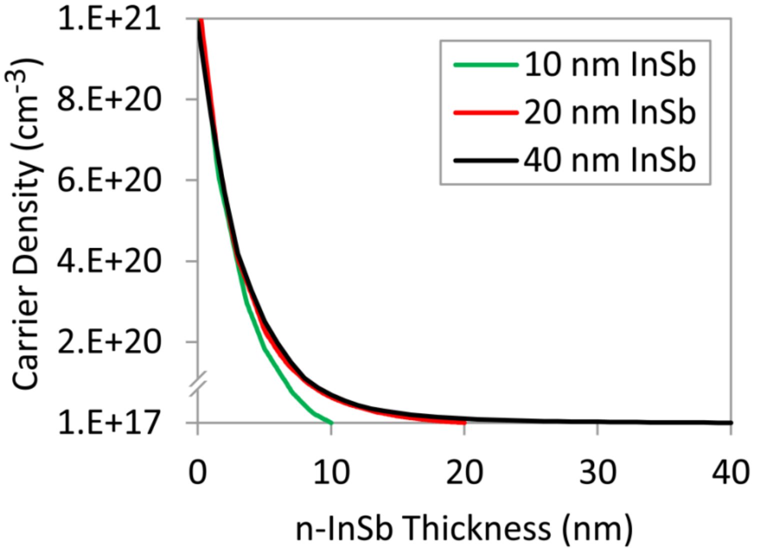

Figure S1. Variation of carrier density (with a -50 V bias) inside the InSb layer for three devices with InSb thicknesses of 10 nm, 20 nm, and 40 nm.
